# Comparison of Five-Year Survival Rate Between Black and White Children With Acute Lymphoblastic Leukemia

**DOI:** 10.7759/cureus.11797

**Published:** 2020-11-30

**Authors:** Courtney Bryant, Mackenzie Mayhew, Jorge Fleites, Juan Lozano, John M Saunders

**Affiliations:** 1 Pediatric Oncology, Florida International University, Herbert Wertheim College of Medicine, Miami, USA; 2 Translational Medicine, Florida International University, Herbert Wertheim College of Medicine, Miami, USA; 3 Pediatric Medicine, Florida International University, Herbert Wertheim College of Medicine, Miami, USA

**Keywords:** acute lymphoblastic leukemia, race, survival, cancer, acute lymphoblastic leukemia (all)

## Abstract

Introduction

Despite improvements in the prognosis of acute lymphoblastic leukemia (ALL), it is still the most common childhood cancer. The goal of this study was to investigate if there was a significant difference in the five-year survival between Black and White children with ALL, specifically up to the year 2016 which has not been researched.

Methods

A retrospective cohort study of Black and White children diagnosed with ALL between 1975 and 2016 was carried out using the Surveillance, Epidemiology, and End Results (SEER) Program database. Children aged 0-19 were separated into Black or White, and then survival analysis was used to compare five-year survival. A multivariate cox regression analysis was carried out to determine the association between race and five-year survival with ALL.

Results

Our sample included 17,663 cases consisting of 16,238 White children and 1,425 Black children. White children had a significantly increased five-year mortality survival when compared to Black children. Upon using multivariate cox regression analysis, both unadjusted and adjusted models showed a significantly higher risk of death in Black children when compared to White children.

Conclusions

Our study found that there is a significant difference in the five-year survival between Black and White children diagnosed with ALL. The difference in survival persists even when controlling for sex, age at diagnosis, year of diagnosis, and histology. Future studies should be carried out to control for more confounders that the SEER database is unable to control for.

## Introduction

Although there have been major improvements in the prognosis of acute lymphoblastic leukemia (ALL), it is still the most common childhood cancer [1]. ALL can arise through either a T-cell or B-cell lineage [2]. Many different risk factors contribute to this childhood cancer, and although the overall survival rate is now 90%, it is still the most frequent cause of death among this cohort [2]. Factors associated with poor prognosis in ALL include male gender, age at diagnosis (<one year or > 10 years), chromosomal abnormalities, hypodiploidy, T-cell immunophenotype, and peripheral white blood cell count greater than 50000/μL [3]. The clinical trials that use this information to provide risk-adaptive care succeeded in decreasing mortality and toxicity among children with ALL as well as increase their relapse-free and overall survival[1].

Whether race/ethnicity affects the survival rate of ALL and by how much has been the topic of the question with mixed results among different studies and clinical centers [3]. The St. Jude’s Research Hospital performed a study in which they used a 30-year study period and found that when looking at the early treatment era, there was a significant difference in the five-year survival rates of Blacks and Whites. When looking at the recent treatment era, however, they found that there was no significant difference [4]. Another study looking at the B-precursor ALL found an excess mortality rate of 42% in Black children compared with White children after adjusting for other factors such as age, sex, the era of treatment, leukocyte count, and leukemia blast cell ploidy [5].

Overall, several studies have looked at the differences in the five-year survival rate of Black and White children with ALL; however, confounding variables such as social determinants of health and access to care are also important factors that many studies have failed to assess. Our study explored the survival rate differences between Black and White children diagnosed with ALL to make steps towards providing equal and unbiased care for every child diagnosed. We also wanted to determine if the gap in survival between races changed over time.

## Materials and methods

Study design

This was a retrospective cohort study using the Surveillance, Epidemiology, and End Results (SEER) Program database, a program of the National Cancer Institute (NCI) created in 1973 [6]. It looks at cancer incidence and survival among patients in the United States. Currently, the database includes information on 34.6% of the US population using population-based cancer registries. The program is updated annually and collects data on patient demographics, primary tumor site, tumor morphology and stage at diagnosis, the first course of treatment, and follow-up for vital status. The mortality date received by the SEER program originates from the National Center for Health Statistics. To ensure that data quality remains high, the NCI staff work with the North American Association of Cancer Registries (NAACR) to help all state registries with their data content and compatibility for acceptable pooling of data and improvement in national estimates. Since we used de-identified data from the SEER database, the study is considered exempt from full review from the institutional review board.

Population and sample

Our study included children in the age range of 0-19 years old who were diagnosed with ALL between 1975 and 2016. We chose this age range because it includes more cases of ALL in this cohort and there were many previous studies to compare our findings.

Variables

The independent variable of this study (exposure) was race. Although the SEER database has four categories for race, White, Black, American Indian/Alaskan Native, and Asian or Pacific, for this particular study we only looked at White and Black.

The dependent variable of the study (outcome) is the true five-year survival rate measured by survival analysis.

Confounders accounted for were: sex, age in years at diagnosis (separated into four age groups: 1-4, 5-9, 10-14, 15-19), year of diagnosis between 1975 and 2016, and histology type based on histology codes as per the third edition of International Classification of Diseases for Oncology (ICD-O-3).

Analysis

Survival analysis was used to assess the two groups’ five-year survival rate. Kaplan-Meier survival curves were plotted according to race. Also, both unadjusted and adjusted Cox regression models were used to determine if there were any associations between race and survival. Upon completion, hazard ratios and their corresponding 95% intervals were calculated allowing for proportional hazard assumptions to be tested graphically.

## Results

Sample characteristics

**Figure 1:**
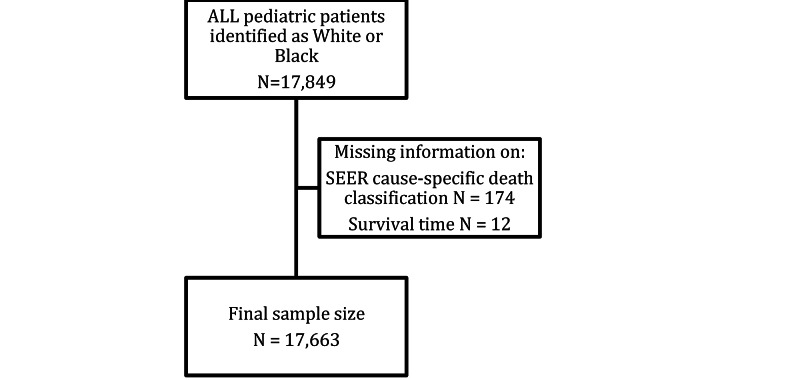
Summary of the inclusion and exclusion of cases from the SEER database search

After using the SEER database to search for Black and White pediatric patients diagnosed with ALL in the study period, 17,663 cases met our inclusion criteria (Figure 1). Of the cases included, there were 16,238 (91.9%) White children and 1,425 (8.1%) Black children. Table 1 summarizes the baseline characteristics of the cohorts. There was no significant difference in sex distribution between both groups (p=0.83). There was a significant difference in the distribution of cases within the age groups, with slightly more children aged 1-4 years among Whites than in Blacks (47.7% vs 41.5%, respectively; p<.001). There was no significant difference between the two groups of children on the year of diagnosis (p=0.22). B-cell subtype of leukemia made up the majority of the cases in histology for both groups but was significantly more frequent among White than in Black patients (95.1% vs 89.7%; p<.001).

**Table 1:** Baseline characteristics of the study population of 17,663 children diagnosed with acute lymphoblastic leukemia ICD-O-3 codes included in histology: ✝: 9811/3 – B lymphoblastic leukemia/lymphoma, NOS; 9812/3 – Leukemia/lymphoma with t(9;22)(q34;q11.2);BCR-ABL; 9813/3 – Leukemia/lymphoma with t(v;11q23);MLL rearranged; 9814/3 – Leukemia/lymphoma with t(12;21)(p13;q22);TEL-AML1(ETV6-RUNX1); 9815/3 – B lymphoblastic leukemia/lymphoma with hyperdiploidy; 9816/3 – Leukemia/lymphoma with hypodiploidy (hypodiploid ALL); 9817/3 – B lymphoblastic leukemia/lymphoma with t(5;14)(q31;q32);IL3-IGH; 9818/3 – Leukemia/lymphoma with t(1;19)(q23;p13.3); E2A PBX1 (TCF3 PBX1); 9826/3 – Burkitt cell leukemia; 9835/3 – Precursor cell lymphoblastic leukemia, NOS; 9836/3 – Precursor B-cell lymphoblastic leukemia, ◊: 9837/3 – T lymphoblastic leukemia/lymphoma

Table 1. Baseline characteristics of the study population of 17,663 children diagnosed with acute lymphoblastic leukemia					
Characteristics	Race				p-value
	White		Black		
	N	%	N	%	
Sex					0.83
Male	9198	56.6	803	56.4	
Female	7040	43.4	622	43.7	
Age (years)					<0.001
<1	465	2.9	62	4.4	
1-4	7266	44.8	529	37.1	
5-9	4039	24.9	360	25.3	
10-14	2455	15.1	297	20.8	
15-19	2013	12.4	177	12.4	
Year					0.219
1975-1984	1392	8.6	109	7.7	
1985-1994	1930	11.9	169	11.9	
1995-2004	4765	29.3	394	27.7	
2005-2016	8151	50.2	753	52.8	
Histology					<0.001
B-cell^✝^	15446	95.1	1278	89.7	
T-cell^◊^	792	4.9	147	10.3	

Survival analysis

**Figure 2:**
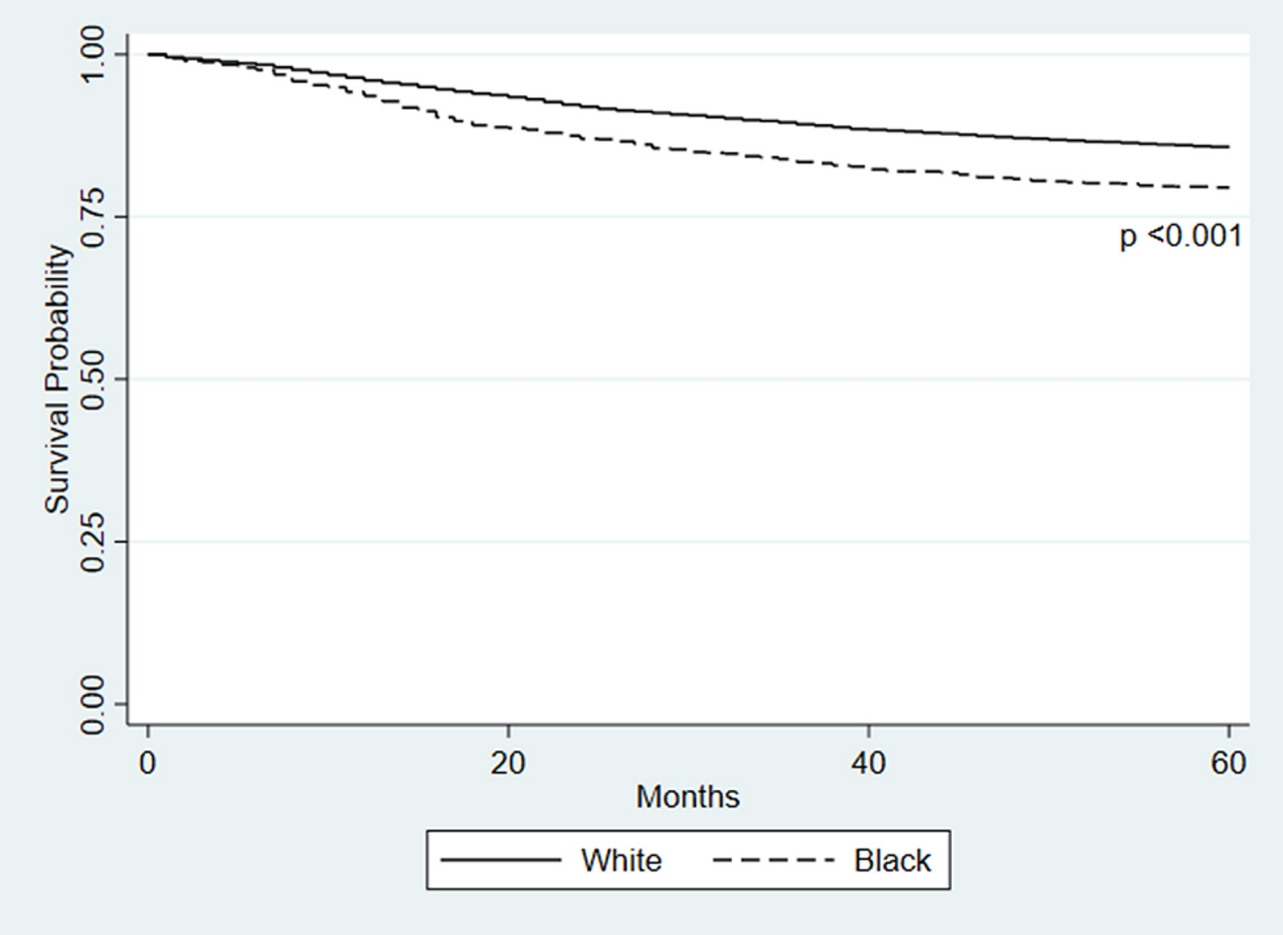
Kaplan-Meier survival probability of children according to race

Overall, White children had a significantly increased five-year survival when compared to Black children (p<0.001). In addition, survival is significantly increased in White children when compared to Black children, as seen in the Kaplan-Meier curve in Figure 2. Furthermore, there were significant differences (p<0.001) found in the survival according to sex, age, and year of diagnosis (Table 2). Females had an increased survival rate when compared to males. Children between 1-9 years of age had increased survival compared to the other age subgroups. Lastly, both groups of children diagnosed with ALL between 1995 and 2016 had increased survival compared to the children diagnosed between 1975 and 1994.

**Table 2:** Survival probabilities among the study population of 17,663 children diagnosed with acute lymphoblastic leukemia ICD-O-3 codes included in histology: ✝: 9811/3 – B lymphoblastic leukemia/lymphoma, NOS; 9812/3 – Leukemia/lymphoma with t(9;22)(q34;q11.2);BCR-ABL; 9813/3 – Leukemia/lymphoma with t(v;11q23);MLL rearranged; 9814/3 – Leukemia/lymphoma with t(12;21)(p13;q22);TEL-AML1(ETV6-RUNX1); 9815/3 – B lymphoblastic leukemia/lymphoma with hyperdiploidy; 9816/3 – Leukemia/lymphoma with hypodiploidy (hypodiploid ALL); 9817/3 – B lymphoblastic leukemia/lymphoma with t(5;14)(q31;q32);IL3-IGH; 9818/3 – Leukemia/lymphoma with t(1;19)(q23;p13.3); E2A PBX1 (TCF3 PBX1); 9826/3 – Burkitt cell leukemia; 9835/3 – Precursor cell lymphoblastic leukemia, NOS; 9836/3 – Precursor B-cell lymphoblastic leukemia, ◊: 9837/3 – T lymphoblastic leukemia/lymphoma

Table 2. Survival probabilities among the study population of 17,663 children diagnosed with acute lymphoblastic leukemia				
Characteristics	Expected	Observed	Log Rank X^2 ^test	p-value
Race			41.24	<0.001
White	2115.5	2033		
Black	179.5	262		
Sex			40.01	<0.001
Male	1291.9	1442		
Female	1003.1	853		
Age (years)			1314.7	<0.001
<1	49.7	198		
1-4	1065.7	561		
5-9	587.3	420		
10-14	341.2	513		
15-19	251.1	603		
Year			709.3	<0.001
1975-1984	189.2	512		
1985-1994	294.7	392		
1995-2004	754.2	687		
2005-2016	1057	704		
Histology			1.63	0.201
B-cell^✝^	2187	2174		
T-cell^◊^	108.1	121		

Multivariate cox regression analysis

To determine the potential confounders included in the Cox regression, collinearity between each was tested. No variables demonstrated collinearity, thus, sex, age at diagnosis, year of diagnosis, and histology were all included in the analysis.

Both unadjusted and adjusted models showed a significantly higher risk of death in Black children as compared to White children (Table 3). The unadjusted risk of death was 52% greater in Black children (hazard ratio (HR) 1.52, 95% CI 1.34 to 1.73; P<0.001). When adjusting for sex, age at diagnosis, year of diagnosis, and histology, Black children had a 45% higher hazard of death than did White children (adjusted HR 1.45, 95% CI 1.28 to 1.66; P0<.001). While the risk of death decreased when adjusting for confounders, Black children still have a significantly greater risk of death. The female children had a lower risk of death than male children in both unadjusted and adjusted models. All age groups had a significantly lower risk of death than children in the <1 year age group in both unadjusted and adjusted models. There was a significantly lower risk of death in the time period of 2005-2016 than in the decades before, both unadjusted and adjusted models. When unadjusted, histology was not significantly associated with differences in survival (P=.202), but when adjusted, it showed a significantly higher risk of death in the T-cell lineage (aHR 1.22, 95% CI 1.01 to 1.48; P<0.05) than the B-cell lineage.

The findings of the analysis looking at the gap in survival between the two races along the four decades of observation are depicted in Table 4. Despite some trends towards improvement, survival was significantly lower for Black children along all four periods of observation. Adjusted hazard ratios ranged from 1.9 (95% CI 1.4-2.5, p<0.001) and 1.3 (95% CI 1.0-1.6, p=0.03), showing that the inequalities persist throughout time.

**Table 3:** Multivariate Cox regression with unadjusted and adjusted associations between acute lymphoblastic leukemia and race, sex, age at diagnosis, year of diagnosis, and histology Abbreviations: CI, confidence interval; HR, hazard ratio. ICD-O-3 codes included in histology: ✝: 9811/3 – B lymphoblastic leukemia/lymphoma, NOS; 9812/3 – Leukemia/lymphoma with t(9;22)(q34;q11.2);BCR-ABL; 9813/3 – Leukemia/lymphoma with t(v;11q23);MLL rearranged; 9814/3 – Leukemia/lymphoma with t(12;21)(p13;q22);TEL-AML1(ETV6-RUNX1); 9815/3 – B lymphoblastic leukemia/lymphoma with hyperdiploidy; 9816/3 – Leukemia/lymphoma with hypodiploidy (hypodiploid ALL); 9817/3 – B lymphoblastic leukemia/lymphoma with t(5;14)(q31;q32);IL3-IGH; 9818/3 – Leukemia/lymphoma with t(1;19)(q23;p13.3); E2A PBX1 (TCF3 PBX1); 9826/3 – Burkitt cell leukemia; 9835/3 – Precursor cell lymphoblastic leukemia, NOS; 9836/3 – Precursor B-cell lymphoblastic leukemia, ◊: 9837/3 – T lymphoblastic leukemia/lymphoma

Table 3. Multivariate Cox Regression with Unadjusted and Adjusted associations between acute lymphoblastic leukemia and race, sex, age at diagnosis, year of diagnosis, and histology.				
Characteristics	Unadjusted		Adjusted	
	HR (95% CI)	p-value	HR (95% CI)	p-value
Race				
White	reference		reference	
Black	1.52 (1.34-1.73)	<0.001	1.45 (1.28-1.66)	<0.001
Sex				
Male	reference		reference	
Female	0.76 (0.70-0.83)	<0.001	0.82 (0.75-0.89)	<0.001
Age (years)				
<1	reference		reference	
1-4	0.13 (0.11-0.15)	<0.001	0.13 (0.11-0.15)	<0.001
5-9	0.18 (0.15-0.21)	<0.001	0.18 (0.15-0.21)	<0.001
10-14	0.38 (0.32-0.44)	<0.001	0.37 (0.31-0.44)	<0.001
15-19	0.60 (0.51-0.71)	<0.001	0.61 (0.52-0.71)	<0.001
Year				
1975-1984	4.07 (3.63-4.56)	<0.001	4.43 (3.94-4.98)	<0.001
1985-1994	2.00 (1.77-2.26)	<0.001	2.24 (1.98-2.54)	<0.001
1995-2004	1.37 (1.23-1.52)	<0.001	1.47 (1.32-1.63)	<0.001
2005-2016	reference		reference	
Histology				
B-cell^✝^	reference		reference	
T-cell^◊^	1.13 (0.94-1.35)	0.202	1.22 (1.01-1.48)	<0.05

**Table 4:** Adjusted hazard ratios of White and Black children with acute lymphoblastic leukemia stratified by decade Abbreviations: CI, confidence interval; HR, hazard ratio

Table 4. Adjusted hazard ratios of White and Black children with acute lymphoblastic leukemia stratified by decade			
Characteristics		Adjusted	
		HR (95% CI)	p-value
1975-1984			
White	Reference		
Black	1.7 (1.3-2.2)		<0.001
1985-1994			
White	Reference		
Black	1.9 (1.4-2.5)		<0.001
1995-2004			
White	Reference		
Black	1.4 (1.1-1.7)		0.017
2005-2016			
White	Reference		
Black	1.3 (1.0-1.6)		0.033

## Discussion

ALL continues to be the most common childhood cancer [1]. Treatment for ALL has made many advances throughout the years leading to a survival rate of over 90% in children. Many past studies have contributed to the high survival rate through research on risk and prognostic factors. Our primary goal of this study was to compare the survival of White and Black children to expose any inequalities that still exist and move closer to bridging the gap in survival between the two races.

This study looked at White and Black children ages 0-19 years old who were diagnosed with ALL. We used the SEER database looking at all relevant diagnoses from the years 1975-2016, excluding only those with insufficient data about survival. After controlling for confounders we found a statistically significant difference in the survival rates between the two races. In addition, we also found that among both races females had an increased survival rate. The age group 1-4 years also had the most cases of ALL. Our findings were congruent with other studies in the past which found that White children have a higher five-year survival rate compared to Black children [1,3-5,7-12]. Between 1995-2016 we found that there was the highest five-year survival rate among both races compared to other years possibly due to the advances in treatment and research on this type of cancer.

Confounders for this study were chosen based on previous studies [1,3-5,7-12]. However, none of these studies looked at data past the year 2009. Since our study includes patients through 2016, it allows for more recent, modern advances and survival data to be taken into account. Due to the fact that we used the SEER database, there were some confounders we were not able to assess because the database did not have these variables. These include socioeconomic status, access to healthcare, genetic risk factors, other comorbidities, disease-specific treatment and relapse data. One study performed by Pui et al. looked at two different populations, one from the SEER database and another from the St. Jude’s Research Hospital [11]. This study was unique in that St. Jude’s Research Hospital gives comprehensive care to every patient regardless of their ability to pay. Although this study only looked at data up to 2007, they found that when access to care was controlled for there were no significant differences in survival rates among Black and White children. Although our study could not control for this confounder, we did find that the survival rate between the two races continues to narrow as advances in treatment continue to be made. Additionally, we found that the difference in survival begins between about 12 to 18 months. Given the findings of the Pui et al. study [11] and that the typical treatment of ALL has three phases [13] - remission induction, consolidation/intensification, maintenance - with the first and second phases lasting between six and eight months [13], we can reasonably postulate that access to care and other socioeconomic issues contribute to the difference in five-year survival between Black and White children.

The strengths of this study include the fact that it was a retrospective study allowing it to be performed quickly and inexpensively. We used a de-identified database giving few ethical issues and allowing us to bypass IRB. However, there were some limitations. For example, the inability to control for access to care and take into account other confounders limited our study. In addition, due to the type of cancer we researched, the grade could not be accounted for either. Instead, histology type was used to parallel the reasoning behind this variable. Therefore, our study shows that, despite some improvement in the last four decades, inequalities in survival have persisted for a long time.

## Conclusions

In conclusion, our study supported that there continues to be a significant difference in the five-year survival rate between Black and White children diagnosed with ALL. Additionally, the rate at which children die is significantly higher in Black children. Sex, age, and year of diagnosis are important confounding variables, but even when controlled for, did not eliminate the difference in survival rates. Future research studies should be done to control for more confounders that were unable to be controlled for using the SEER database. Therefore, studies should also attempt to build off of St. Jude’s Research Hospital to look at the survival rates of different pediatric populations while specifically controlling for access to care.
